# Prevalence of community health-promoting practices in Singapore

**DOI:** 10.1093/heapro/dax101

**Published:** 2017-12-27

**Authors:** Manimegalai Kailasam, Yin Maw Hsann, Priyanka Vankayalapati, Kok Soong Yang

**Affiliations:** Department of Epidemiology, Ng Teng Fong General Hospital, 1 Jurong East Street 21, Singapore 609606, Singapore

**Keywords:** community health promotion, health-promoting environments, access, composite score, residential

## Abstract

Both living and working environments have a substantial influence on promoting healthy living habits. A holistic and accurate assessment of the community health-promoting practices is important to identify gaps and to make continuous, tangible improvements. The aim of the study is to assess the prevalence of the Singapore community health-promoting practices. The community health-promoting practices in all residential zones of an electoral constituency were assessed based on a composite health promotion scoring system comprising of 44 measurable elements under the 5 domains of community support and resources; healthy behaviours; chronic conditions; mental health; and common medical emergencies. An alphabetical grading system was used based on the score ranges: grade ‘A’ (75% and above), grade ‘B’ (60% to below 75%), grade ‘C’ (50% to below 60%) and grade ‘D’ (below 50%). The community health-promoting practices were graded ‘D’ with an overall average score of 41%. The constituency achieved grade ‘C’ (59%) for mental health domain and grade ‘B’ (72%) for common medical emergencies. The health-promoting practices for the other domains were graded ‘D’ (<50%) except for healthy behaviour (physical activity) sub-domain which achieved grade ‘B’ (65%). Significant gaps were identified in the community health-promoting practices. The residential zones may benefit from the scoring system to identify gaps and prioritize high-impact strategies to improve their health practices.

## INTRODUCTION

Like most developed countries, the Singapore Burden of Disease Study 2010 identified the country’s three leading disease burden to be ischaemic heart disease, diabetes mellitus and stroke (Singapore Ministry of Health, 2014). These diseases also impose a severe economic burden in terms of lost productivity and unsustainable medical costs. Diabetes alone cost Singapore more than $1 billion in 2010, and this has been predicted to soar beyond $2.5 billion by 2050 (Png *et al.*, 2016).

It has been estimated that 60–95% of the risks are attributed to potentially modifiable lifestyle and behaviour risk factors (Yusuf *et al.*, 2004; Chiuve *et al.*, 2011). Several studies identify that an environment supporting health-promoting behaviours is more likely to enable individuals to adopt and sustain healthy lifestyles by making healthy living more accessible, natural and effortless (Abraham *et al.*, 2010; Sallis and Glanz, 2009). For instance, adequate safe playgrounds and green spaces in the environment have been identified to play an integral role in encouraging physical activity among residents (Perdue *et al.*, 2003; Sallis *et al.*, 2009). Research conducted in the workplace settings have shown that such an environment is associated with lower medical and absenteeism costs and hence results in substantial cost savings (Baicker *et al.*, 2010; Chapman, 2005).

Composite measures, which combine multiple performance indicators using a predetermined weighting methodology to produce a single score (Austin *et al.*, 2014; US Department of Health and Human Services, 2017) are a means of standardized assessment and easy interpretation. Such measures have been widely employed in assessing clinical quality and safety and hospital performances (Shwartz *et al*, 2015; Austin *et al.*, 2014). The U.S. CDC Worksite Health Scorecard and the HERO Scorecard are the examples of such instruments developed to help employers in assessing their health promotion and wellbeing practices in workplaces (Torres, 2011; Centers for Disease Control and Prevention, 2014). These standardized tools serve to assess the prevalence of health promotion practices in the workplace, identify gaps and help to prioritize high-impact strategies to prevent chronic diseases such as heart disease, stroke and related conditions. Some studies have even found that workplace health promotion practices are predictive of healthcare cost trends for the employers (Goetzel *et al.*, 2014).

Although there are some existing objective measures that quantify health promotion in the community, they are limited to the assessment of only a few specific niche areas. For instance, the walkability integrated index is only useful for evaluating and operationalising walkability to enable residents to be physically active (Frank *et al.*, 2010). There are no known composite measures to comprehensively evaluate the different aspects of health-promoting programmes in the residential community currently based on existing health literature. Identifying the prevalence is important to assess the need for and prioritise improvements in the health-promoting practices.

In this study, we aim to determine the prevalence of health-promoting practices in the residential zones through a composite health promotion scoring system developed as a standardised means of assessment. Residential zones are defined as community sub-divisions within an electoral constituency and each residential zone is served by a residents’ committee whose functions are to promote neighbourliness, racial harmony and community cohesiveness amongst residents within their respective zones in the public housing estates. Run by residents for residents, the residents’ committees work closely with various government agencies to improve the physical environment and safety of their respective precincts.

## METHODS

### Study sample

A purposive sample of an electoral constituency with seven residential zones in the west region of Singapore was selected for the pilot. All seven residential zones were public housing neighbourhoods and comprised of Housing and Development Board (HDB) flats inhabited by about 80% of the Singapore population (Singapore Housing and Development Board, 2015). Each housing block may comprise of 1 to 5 room HDB apartments. Private condominiums and landed properties where a minority of the population belonging to the higher socio-economic status stayed were excluded. With the implementation of an ethnic integration policy in Singapore, racial distribution was similar in the different HDB bocks and neighbourhoods (Singapore Housing and Development Board, 2015). The pilot zones also had a similar age group distribution as that of the national population. Therefore, the sample was considered reasonably representative of the Singapore residential community.

### Development of the composite score

The authors reviewed existing literature on health promotion to identify elements, including interventions, pertinent to changing individual lifestyle and health behaviour. This list was further reviewed to select those elements that were relevant to a residential community. In addition, national health promotion guidelines were incorporated to suit the local context. Forty-four measurable elements were identified to be included in the composite health promotion score and are listed in Table 1. Similar elements were grouped together under the same theme or domain. A total of five domains were formed—Community Support and Resources, Healthy Behaviour, Chronic Conditions, Mental Health and Common Medical Emergencies. The measurable elements under the domain of Healthy Behaviour were further classified into four subdomains—Physical Activity, Healthy Eating, Smoking Prevention and Weight Management. These themes were in accordance with those areas addressed in health promotion programmes in Singapore (https://www.hpb.gov.sg) and other countries (Centers for Disease Control and Prevention, 2014). The face validity of the scoring system was then assessed by public health experts from a national health agency (Health Promotion Board, Regional Health & Community Outreach Division) and an academic professor from the Saw Swee Hock School of Public Health, National University of Singapore. Their comments and suggestions were then used to fine-tune the scoring system.

**Table 1:** List of measurable elements included in composite health promotion score

Measurable element	Evidence score	Impact score	Weightage
*Community support and resources (CSR)*			
CSR-1: Health promotion committee	2	2	2
CSR-2: Designated health promotion officer	2	2	2
CSR-3: Dedicated funds for health promotion programmes	2	2	2
CSR-4: Annual objectives for health promotion	2	2	2
CSR-5: Publicity for health promotion programmes	2	2	2
CSR-6: Leaders’ commitment to health and participation in activities	2	3	2
CSR-7: Incentives for participation	2	2	2
CSR-8: Age appropriate competitions to improve health	2	3	2
CSR-9: Literacy/Culture appropriate health promotion programmes	3	3	3
CSR-10: Feedback for health promotion programmes	1	2	1
CSR-11: Promotion of other national public health initiatives/events	2	2	2
*Healthy behaviour (HB)*			
*Physical activity*			
HB-1: Adequate exercise facilities	3	3	3
HB-2: Exercise facilities’ appropriateness for population profile	3	3	3
HB-3: Variety of exercise equipment/facilities	2	3	2
HB-4: Behavioural nudges to encourage residents to be active (e.g. use stairs instead of lift)	3	3	3
HB-5: Regular group physical activity programmes appropriate for population profile	3	3	3
*Healthy eating*			
HB-6: Healthier eateries	3	3	3
HB-7: Access to water or sale of cheaper bottled water	2	2	2
HB-8: Provision of healthier food/beverage during constituency events	1	1	1
HB-9: Training for skills related to healthy eating	2	2	2
*Smoking prevention*			
HB-10: No smoking fine signs displayed in common areas of high non-compliance to smoking prohibition	3	3	3
HB-11: Voluntary creation of a smoke free zone	3	3	3
HB-12: Promoting tobacco cessation programmes	3	3	3
*Weight management*			
HB-13: Promoting regular self-evaluation of weight/BMI	2	2	2
HB-14: Promoting public weight management programmes	1	1	1
HB-15: Conducting weight management programmes	3	3	3
*Chronic conditions (CC)*			
CC-1: Free/subsidized health screening to detect chronic conditions	3	3	3
CC-2: Free/subsidized health screening to detect common cancers	3	3	3
CC-3: Functional screening to detect age-related functional decline	3	3	3
CC-4: Screened residents undergo appropriate clinical follow up	3	3	3
CC-5: Self-management programmes for chronic conditions	3	3	3
CC-6: Talks and training to caregivers of elderly	2	2	2
CC-7: Health fairs	1	3	2
*Mental health (MH)*			
MH-1: Social events/activities for social networking and bonding	1	1	1
MH-2: Support system to provide social and emotional support	2	3	2
MH-3: Support system to provide physical support	2	3	2
MH-4: Support system to provide tangible assistance	2	3	2
MH-5: Age appropriate life skills training programmes	2	2	2
*Common medical emergencies (CME)*			
CME-1: Warning signs of heart attack/stroke displayed in common areas	2	1	1
CME-2: Directions on what to do if symptoms are present displayed	2	1	1
CME-3: AED equipped in community centre	3	3	3
CME-4: Personnel trained in AED available in the community centre at all times	2	2	2
CME-5: AEDs are routinely maintained and tested	2	1	1
CME-6: Access to training on CPR/AED to residents	3	3	3

### Weighting methodology

Two criteria were identified to assign weights to each measurable item: strength of evidence and impact. The rating systems for both criteria were adapted from the CDC Worksite Health Scorecard (Centers for Disease Control and Prevention, 2014) and outlined below:

- Rating system for strength of evidence

Rating score = 1 (Weak): Relationship is supported by fragmentary, non-experimental or poor operationalized studies

Rating score = 2 (Suggestive): Relationship is supported by ≥ 2 studies, such as pre-post evaluations or quasi-experimental studies

Rating score = 3 (Strong): Relationship is supported by ≥1 well-designed randomized controlled trials or ≥3 well-designed quasi-experimental studies or systematic reviews

- Rating system for impact

Rating score = 1 (Small): Experts debate on the plausibility of the causal impact

Rating score = 2 (Sufficient): Most experts believe causal impact is plausible and consistent with knowledge in relevant areas

Rating score = 3 (Large): Little or no debate on causal impact

The criteria of strength of evidence and impact were assessed based on a comprehensive literature review as well as publicly available government reports of health promotion interventions (US Department of Health and Human Services, 2017).

### Scoring methodology

Similar to the weighting methodology, the calculation of the composite health promotion score was also adapted from the U.S. CDC Worksite Health Scorecard. The weighted score for each measurable item was calculated as a sum of the rating scores for “strength of evidence” and “impact” from which the weightage was derived. Hence, the weightage is an indicator of the measurable item’s impact on health outcomes based on the strength of evidence found in existing literature (Centers for Disease Control and Prevention, 2014).

**Table:** 

Strength of Evidence +	Impact =	Weighted Score =	Weightage
1=Weak	1=Small	Total points=2, 3; Value=1	1
2=Suggestive	2=Sufficient	Total points=4, 5; Value=2	2
3=Strong	3=Large	Total points=6; Value =3	3

The overall composite health promotion score calculated for each residential zone was then computed as a percentage of the overall maximum score and was used to grade the performance of the health-promoting environment of the residential zone under assessment. Guidelines were created for objective scoring. An alphabetical grading system was used based on the score ranges: grade ‘A’ (75% and above), grade ‘B’ (60% to below 75%), grade ‘C’ (50% to below 60%) and grade ‘D’ (below 50%).

A total of three raters were involved in the scoring and they took turns such that each residential zone was rated by any two of them. All the raters were medical graduates with a master’s degree in public health with at least 2 years’ working experience as a hospital epidemiologist. The scoring methodology and guidelines for the score card were modelled and adapted from international hospital quality accreditation and audit standards. Weighted scores for all the zones were identified based on these guidelines. The scoring methodology was developed by a senior public health consultant with relevant work experience in healthcare system quality accreditation and audit standards, and was responsible for training of the three raters. Each rater received a total of 3 hours in training to familiarise themselves with the survey tools and scoring methods.

### Assessment methodology

The assessment of each zone was conducted in two parts. The first part of the assessment involved an interview with the Chairman and at least two other relevant residents’ committee members to obtain information about the zone’s health programmes and management practices. The second part included a site visit within the boundaries of the residential zone to assess the built environment and its facilities e.g. fitness corners, eateries such as restaurants, food courts etc.

## RESULTS

### Constituency characteristics

The composite health promotion score developed was piloted in an electoral constituency in the west region of Singapore. The electoral constituency comprised of seven residential zones with 130 housing blocks with an electorate population of about 22 000. Each zone is managed by their respective residents’ committee with the elected Member of Parliament as their grassroots advisor. One of the residential zones, Zone 4, was positioned as a model health-promoting zone and had implemented several health promotion programmes.

### Scores of the residential zones

Tables 2 and 3 provide a summary of the scores for each of the five domains and the overall score based on the epidemiologists’ assessment. The health-promoting practices of the residential zones were graded ‘D’ (below 50%) with an overall score of 41%. For the themes related to health promotion that were assessed, the elements for Mental Health was graded ‘C’ (50% to below 60%) and that of Common Medical Emergencies was graded ‘B’ (60% to below 75%). The performance with regards to the other themes—Community Support and Resources, Healthy Behaviour and Chronic Conditions were graded ‘D’. Among the subdomains under healthy behaviour, elements related to physical activity were graded ‘B’.

**Table 2:** Scores of the residential zones by domain

	Max score	RZ's score, *n* (%)	Grade
Community support & resources	44	18 (40)	D
Healthy behaviour	74	28 (38)	D
Healthy behaviour (physical activity)	28	18 (65)	B
Healthy behaviour (healthy eating)	16	6 (37)	D
Healthy behaviour (smoking prevention)	18	2 (12)	D
Healthy behaviour (weight management)	12	2 (14)	D
Chronic conditions	38	8 (20)	D
Mental health	18	11 (59)	C
Common medical emergencies	22	16 (72)	B
Overall	196	80 (41)	D

RZ- Residential Zone

**Table 3:** Summary of scores by zone and domain

Domain	Zone 1	Zone 2	Zone 3	Zone 4	Zone 5	Zone 6	Zone 7	P-value
CSR	15	13	15	30	21	15	15	0.00005
HB	31	20	25	36	35	17	31	0.02
HB (PA)	19	16	19	19	19	13	22	0.47
HB (HE)	4	4	6	7	8	4	8	0.16
HB (SP)	6	0	0	3	6	0	0	0.47
HB (WM)	2	0	0	7	2	0	1	0.00002
CC	2	2	14	21	0	4	10	0.0009
MH	10	10	10	12	10	12	10	0.004
CME	15	15	15	18	12	18	18	0.04
Overall	73	60	79	117	78	66	84	0.00007

CSR, Community support and resources; HB, Healthy Behaviour; HB (PA), Physical Activity; HB (HE), Healthy Eating; HB (SP), Smoking Prevention; HB (WM), Weight Management; CC, Chronic conditions; MH, Mental Health; CME, Common Medical Emergencies

At the zonal level, the overall composite % score ranged from 31% (60/196) to 60% (117/196) of the maximum overall score. All except Zone 4 was graded ‘D’ in terms of their overall health-promoting performance. Zone 4 had the best performance and was graded as ‘B’ based on the assessment scheme. The composite score ranged from 0% for the chronic conditions domain to 82% for common medical emergencies. A large variation in performance was seen in the chronic conditions domain where the scores ranged from 0 in zone 5 to 55% in Zone 4. When compared to other zones, Zone 4 scored significantly higher (*p* ≤ 0.05) overall and for all the specific domains. In contrast, all the other zones fared not significantly different (*p* > 0.05) for subdomains under healthy behaviour except for weight management (Table 3).

## DISCUSSION

This study estimates that the Singapore community health-promoting practices are not adequate. Significant gaps were identified in the areas related to community support & resourcing, efforts to support and nudge the adoption of healthy behaviours as well as in the areas of chronic disease prevention and management. Most residential zones had sufficient initiatives to support physical activities even though they fared poorly under the other domains. This showed that most zones had a tendency to prioritise physical activities as the predominant feature of health promotion.

Zone 4, which had the best grades, had implemented several initiatives to promote community health in collaboration with the Health Promotion Board, and it had been positioned as the model health-promoting zone. This zone was found to be pro-actively sourcing for resources to improve their overall health-promoting practices. The fact that this zone scored better in our assessment could be an indication of the construct validity of our scorecard. But in spite of having better overall scores, Zone 4 did not have adequate practices to support healthy eating and smoking prevention, both of which are also important and holistic healthy behaviour components. There are several strengths associated with this study. This is the first study that assesses the community health-promoting practices in an objective and comprehensive manner. Traditionally, such objective measures were only focussed on specific niche area while this study attempts a holistic assessment. Secondly, the scoring system is based on best practices identified in existing literature and is further emphasized through the weighting and scoring methodologies described. Therefore, it can help to identify gaps in the current health-promoting efforts in the community and serves to easily identify priorities for the grassroots leaders to focus their improvement efforts on. Objective measures are included in the composite score instead of subjective ones, thereby reducing observation biases and increasing internal validity. Thirdly and lastly, the composite scores can possibly drive healthy competition through peer pressure among the residential zones and also help identify and learn best implementation practices from each other, thereby raising the overall healthy promoting performance of all residential communities in Singapore.

The main limitation for this study is that the composite scoring system is specifically developed to suit Singapore’s grassroots community and hence may not be directly suitable in other countries. However, as most of the measurable elements are based on studies conducted in multiple communities, they may be adapted to better suit the local context of different countries. Next, the raters were not blinded in this study, and they were aware of Zone 4’s status as a model health-promoting zone. Nevertheless, scoring guidelines with objective assessment criteria should have negated any significant bias in favour of Zone 4.

The health-promoting practices of the community can be measured using the composite health-promotion scoring system. Inter-rater validity is an important consideration to minimize variations in scores evaluated by different assessors in different residential zones. It is beyond the scope of this paper to describe the validity of the scoring system and the assessment of inter-rater variability. Those elements would be studied separately and published later. The scoring system could be updated periodically when new evidence evolves in the field of community health promotion.

## CONCLUSION

Significant gaps are identified in the Singapore community health-promoting practices. Residential zones that are driven to improve their health-promoting practices can work in collaboration with relevant industry partners and achieve a better health-promoting environment. They can make use of the composite health promotion scoring system to identify the gaps and to prioritize high-impact strategies to improve their health-promoting practices. Even though the composite health promotion scoring system was developed for use in Singapore residential areas, there could be relevance to other urban areas around the world. The demand for better health assessments of residential communities is high and this assessment tool may possibly be adapted to other countries’ context.
